# Discovery of New Phenylacetone Monooxygenase Variants for the Development of Substituted Indigoids through Biocatalysis

**DOI:** 10.3390/ijms232012544

**Published:** 2022-10-19

**Authors:** Nicolás Núñez-Navarro, Javier Salazar Muñoz, Francisco Castillo, César A. Ramírez-Sarmiento, Ignacio Poblete-Castro, Flavia C. Zacconi, Loreto P. Parra

**Affiliations:** 1 Facultad de Química y de Farmacia, Pontificia Universidad Católica de Chile, Santiago 7820436, Chile; 2 Institute for Biological and Medical Engineering, Schools of Engineering, Medicine and Biological Sciences, Pontificia Universidad Católica de Chile, Santiago 7820436, Chile; 3 Department of Chemical and Bioprocesses Engineering, School of Engineering, Pontificia Universidad Católica de Chile, Santiago 7820436, Chile; 4 Center for Nanomedicine, Diagnostic & Drug Development (ND3), Universidad de Talca, Talca 3460000, Chile; 5 ANID—Millennium Science Initiative Program—Millennium Institute for Integrative Biology (iBio), Santiago 8331150, Chile; 6 Biosystems Engineering Laboratory, Department of Chemical and Bioprocess Engineering, Universidad de Santiago de Chile (USACH), Santiago 8350709, Chile

**Keywords:** Baeyer-Villiger monooxygenase, phenylacetone monooxygenase, indigo derivatives, biocatalysis, eco-friendly dye, indole, L-tryptophan

## Abstract

Indigoids are natural pigments obtained from plants by ancient cultures. Romans used them mainly as dyes, whereas Asian cultures applied these compounds as treatment agents for several diseases. In the modern era, the chemical industry has made it possible to identify and develop synthetic routes to obtain them from petroleum derivatives. However, these processes require high temperatures and pressures and large amounts of solvents, acids, and alkali agents. Thus, enzyme engineering and the development of bacteria as whole-cell biocatalysts emerges as a promising green alternative to avoid the use of these hazardous materials and consequently prevent toxic waste generation. In this research, we obtained two novel variants of phenylacetone monooxygenase (PAMO) by iterative saturation mutagenesis. Heterologous expression of these two enzymes, called PAMO_HPCD_ and PAMO_HPED_, in *E. coli* was serendipitously found to produce indigoids. These interesting results encourage us to characterize the thermal stability and enzyme kinetics of these new variants and to evaluate indigo and indirubin production in a whole-cell system by HPLC. The highest yields were obtained with PAMO_HPCD_ supplemented with L-tryptophan, producing ~3000 mg/L indigo and ~130.0 mg/L indirubin. Additionally, both enzymes could oxidize and produce several indigo derivatives from substituted indoles, with PAMO_HPCD_ being able to produce the well-known Tyrian purple. Our results indicate that the PAMO variants described herein have potential application in the textile, pharmaceutics, and semiconductors industries, prompting the use of environmentally friendly strategies to obtain a diverse variety of indigoids.

## 1. Introduction

Indigoid pigments of natural origin have been used for more than 4000 years. In Mediterranean cultures, their main use was in the textile field, and in Indo-Asian cultures, their medicinal properties were exploited [1,2]. At that time, these pigments were initially extracted from plants (*Indigofera tinctoria*, true indigo; *Isatis tinctoria*, woad) or animals (*Hexaplex trunculus*, sea snail). It was not until the end of the 19th century when a rapid expansion in knowledge was generated by the interest of the Asian market in this pigment. Consequently, extraction from natural resources was no longer viable due to the high production costs and the inaccessibility to these starting materials in the required quantities. Thus, the development of a chemical synthesis became necessary.

**Figure 1:**
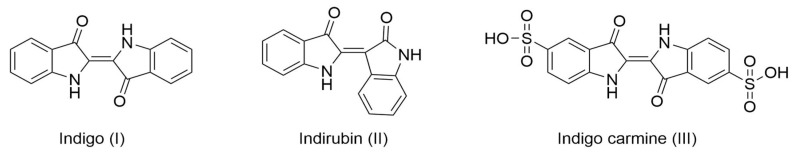
Chemical structure of notable natural indigoids, indigo and indirubin, and synthetic indigo carmine.

It was not until 1883 that BASF (Badische Anilin und SodaFabrik) determined the chemical structure of indigo (Figure 1), enabling the chemical synthesis from fossil-based starting material such as benzene and aniline. After that, in 1897, this company became the major producer of indigo on an industrial level based on fossil feedstocks and agreed to form a business with China, where jackets dyed with natural indigo were traditional clothing items [3].

By 2010, the worldwide production of indigo reached nearly 50,000 tons/year, and the textile industry consumed 95% of the total production for dyeing jeans [4]. The remaining 5% was used as a precursor for dyes in the food and pharmaceutical industries (indigo carmine, FD&C Blue No. 2), mainly as its sulfonic salt, indigo carmine (Figure 1). Additionally, further research has emerged for its application in the development of eco-friendly organic semiconductors [5,6], and has demonstrated its positive effect in traditional Chinese medicine to treat psoriasis and other inflammatory diseases [7,8,9,10].

As an alternative to synthesis based on fossil resources, extensive research has explored the biosynthesis of this pigment [11,12]. Chemical analysis of the biosynthetic routes in woad enabled the identification of intermediates in its formation, such as indoxyl, determining that the main substrate is tryptophan, due to its indole ring [13]. Indoles are compounds containing a benzene ring fused to a five-membered nitrogen-containing pyrrole ring. Once oxidized, indole becomes indoxyl, which in the presence of air oxygen, spontaneously dimerizes into indigo [14]. Another product of tryptophan oxidation, isatin, can also dimerize into another indigoid called indirubin (Figure 1) [15], which has shown several pharmacological activities in traditional Chinese medicine as a treatment for skin diseases (psoriasis, eczema) or systemic inflammatory diseases [9,16].

Some environmental bacteria naturally synthesize indigo from indole only in the presence of aromatic compounds. For instance, *Pseudomonas* sp. HBO1 produced indigo at a yield of 246 mg/L from indole, as it harbors a naphthalene dioxygenase [17]. Likewise, *Pseudomonas putida* synthesized the blue dye by the action of a styrene monooxygenase from indole and *Pseudomonas* sp. PI1 in the presence of phenol [18].

Due to the importance of indigoids for textile and pharmaceutical industries, and looking for environmentally friendly manufacturing strategies, several research groups are working on their production using biocatalytic processes, such as the use of recombinant bacteria, especially in *Escherichia coli*, overexpressing enzymes to catalyze the synthesis of these molecules [14,19,20,21,22,23].

In this work, we describe new variants of an enzyme of the Baeyer–Villiger Monooxygenase (BVMO) family [24], a phenylacetone monooxygenase (PAMO) [25] from the hyperthermophilic bacterium *Thermobifida fusca*, which serendipitously were found to produce indigoids upon recombinant protein overexpression in *E. coli*. While the wild-type enzyme does not accept indole as substrate, these PAMO variants, derived from previous protein engineering works [26,27,28,29], not only produce indigo and indirubin, but also can synthesize several indigoids from alkylated or halogenated indoles as substrate [20,22].

Altogether, this research puts forward the use of BVMOs as greener biocatalytic alternatives to synthesize indigoids, using renewable resources as a starting material and avoiding the use of toxic and contaminant industrial reagents such as aniline or naphthalene.

## 2. Results

### 2.1. Thermostability of PAMO Variants

Two PAMO variants were developed as described in Section 4, and upon inducing protein overexpression in *E. coli* TOP10 cells grown on Terrific Broth (TB) medium were serendipitously found to produce a blue pigmentation (Figure S1), which was later confirmed to correspond to the acceptance of indole as substrate and its conversion into either indigo or indirubin.

**Figure 2:**
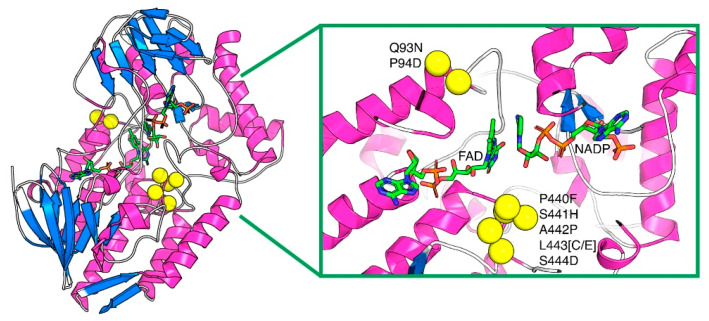
Location of the residue substitutions in the indigoid-producing PAMO_HPCD_ and PAMO_HPED_, mapped onto the structure of PAMO_WT_ (PDB ID: 2YLR). The protein is presented in cartoon representation and the cofactors NADP and FAD are presented in sticks. The residue substitutions, which are in the vicinity of the cofactors within its active site, are shown as yellow spheres. In yellow: substituted amino acids; FAD and NAPD in sticks; and protein loops in blue and pink.

These two new variants are hereafter named PAMO_HPCD_ and PAMO_HPED_ due to their substitutions in residues 441–444 (Figure 2). Substitutions Q93N, P94D, P440F, S441H, A442P, and S444D are common for both, whereas L443 was substituted into cysteine (C) and glutamate (E) for PAMO_HPCD_ and PAMO_HPED_, respectively (Figure S2).

Once overexpressed in *E. coli*, His-tagged wild-type PAMO (PAMO_WT_), PAMO_HPCD_, and PAMO_HPED_ were purified by cell lysis, immobilized metal affinity chromatography (IMAC), and clarified using a centrifugal concentrator of 50 kDa MWCO. Once purified, the enzymes were loaded in an SDS-PAGE, confirming the success and apparent homogeneity of the purification of all enzymes (Figure S3).

Given that PAMO_WT_ is a thermophilic enzyme [25] and that other directed evolution variants of this enzyme harboring four equivalent substitutions out of the seven substitutions present in PAMO_HPCD_ and PAMO_HPED_ show small changes in thermostability [30], we first performed differential scanning calorimetry (DSC) to determine their melting temperature (Tm) and ascertained that the additional substitutions in these variants cause no significant changes in thermal stability in comparison to PAMO_WT_.

The results demonstrate that the seven substitutions in these PAMO variants had no significant effect on the melting temperatures and, therefore, the thermal stability of the modified enzymes. In another study, the Tm for PAMO_WT_ using a differential scanning fluorescence method was 60.5 °C [28], similar to the Tm determined for this enzyme using DSC in this work, corresponding to 60.3 °C. Moreover, the difference in Tm between PAMO_WT_ and the PAMO_HPCD_ and PAMO_HPED_ variants was less than 1 °C (59.7 °C for PAMO_HPCD_ and 59.5 °C for PAMO_HPED_) (Figure S4). However, both variants present a pre-transition process at 51–52 °C compared with the wild-type. This pre-transition state has been reported for other enzyme variants and suggests that the additional mutations in the PAMO_HPCD_ and PAMO_HPED_ could have a detrimental effect on the activity of these enzymes at this temperature range [31].

### 2.2. Structural and Quantitative Analysis of Whole-Cell Biosynthesis of Indigoids

**Figure 3:**
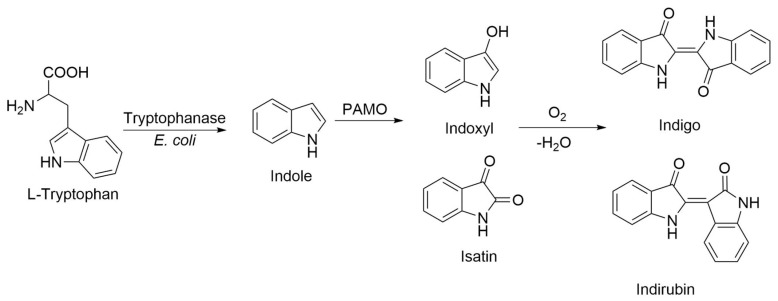
Proposed route of biosynthetic production of indigo in *E. coli* cells overexpressing PAMO_HPCD_ and PAMO_HPED_.

Several elements strongly suggested that the blue pigments observed in *E. coli* cell cultures overexpressing PAMO_HPCD_ and PAMO_HPED_ corresponded to indigoids. First, the pigments were produced in the absence of additional substrates, and the overexpression of PAMO_WT_ in *E. coli* did not result in pigmentation of the cell culture. Second, as *E. coli* naturally converts tryptophan into indole due to the action of a tryptophanase (Figure 3) [32], we also employed this amino acid as a biosynthesis precursor, which did not yield indigo or indirubin. Third, it is important to emphasize that some flavin monooxygenase can oxidize indole and enable the formation of indigo after non-enzymatic dimerization [33]. Fourth, the M446G variant of PAMO_WT_ was described to produce indigo from indole as substrate in kinetic assays using isolated enzymes [26]. Fifth, preliminary assays to determine the presence of indigoids using chloroform extraction from *E. coli* cells overexpressing PAMO_HPCD_ and PAMO_HPED_, followed by silica gel chromatography, enabled the extraction of two pigments of blue and pink coloration, consistent with the production of indigo and indirubin after oxidation of indole into indoxyl and isatin and non-enzymatic dimerization.

**Figure 4:**
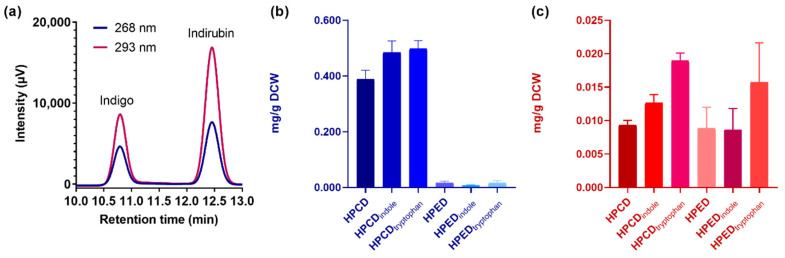
Quantification of indigo and indirubin production in whole-cell biocatalysis using PAMO_HPCD_ and PAMO_HPED_. (**a**) Mixture of calibration standards for indigo and indirubin (5 ppm for each indigoid), measured by absorbance at 268 and 293 nm, demonstrating their sufficient separation in retention time. (**b**) Quantification of indigo production at 268 nm under different cell culture conditions. (**c**) Quantification of indirubin production at 293 nm under different cell culture conditions. Culture conditions: TB alone (HPCD/HPED), TB supplemented with indole 20 μM (HPCD/HPED_indole_), TB supplemented with L-tryptophan 200 μM (HPCD/HPED_tryptophan_).

Based on these results, we quantified the amount of biosynthetically produced indigo and indirubin by HPLC. In these assays, ~5 mL samples obtained from 50 mL cell cultures of *E. coli* TOP10 overexpressing either enzyme and grown in TB medium for 24 h were treated with lysis buffer and subjected to sonication and then to indigoid extraction with ethyl acetate. For quantification, we employed a calibration curve obtained using commercially available and pure indigo and indirubin as standards (Figure S5). Moreover, by injecting a mixture of these calibration standards, we verified that the retention time was sufficiently different for each indigoid to enable their quantification (Figure 4a).

As seen in Figure 4b and Table 1, PAMO_HPCD_ produces ~0.4 mg/g dry cell weight (DCW) of indigo (3000 mg/L), which is 26-fold higher than the production observed for PAMO_HPED_ under the same conditions.

Previous works on indigo biosynthesis by whole-cell biocatalysis using *E. coli* cells overexpressing either a cytochrome P450 monooxygenase from *Streptomyces cattleya* [20] or a flavin monooxygenase *Corynebacterium glutamicum* [34] showed a significant increase in the production of indigo by supplementation of the culture media with tryptophan or indole. Thus, we also performed these experiments on TB medium supplemented with either 20 μM indole or 200 μM L-tryptophan.

A 28% and 25% increase in indigo production for PAMO_HPCD_ was observed when *E. coli* cell cultures were supplemented with L-tryptophan and indole, respectively. PAMO_HPED_ showed no significant changes in indigo production between the different cell culture conditions. For indirubin, both PAMO_HPCD_ and PAMO_HPED_ produced similar amounts (~0.008 mg/g DCW) in the absence of indole or tryptophan (Figure 4c and Table 1). While PAMO_HPED_ showed non-significant differences in indirubin production for all conditions, a statistically significant increase (*p* < 0.0001) of more than 2-fold was recorded for PAMO_HPCD_ upon addition of 200 μM L-tryptophan.

Considering the measured production of indigo per DCW and that approximately 7 g DCW are typically obtained from a 1 L culture of *E. coli* cells in this culture medium overnight the expected production of indigo via whole-cell biocatalysis using PAMO_HPCD_ corresponds to 3.00 g/L of cell culture without requiring supplementation with indole or L-tryptophan. This is 3-fold higher than the production obtained by *E. coli* whole-cell biocatalysis using a flavin monooxygenase from *Methylophaga aminisulfidivorans* for indigo [35]. For indirubin, the production upon supplementation with 200 µM L-tryptophan corresponds to 133.0 mg/L of cell culture, which is similar to the value recently reported for whole-cell biocatalysis using *E. coli* overexpressing an active site variant (R292A) of a BVMO from *Acinetobacter radioresistens* (138 mg/L) [36].

### 2.3. PAMO_HPCD_ and PAMO_HPED_ also Accept Substituted Indoles as Substrates

Beyond the biosynthesis of indigo and indirubin, there is increasing interest in the biocatalytic production of halogenated indigoids. For example, Tyrian purple (6,6′-dibromoindigo), the oldest known purple dye used in imperial clothes thousands of years ago, which is now being employed in semiconductor materials [37], requires 10,000 sea snails that are the natural source to produce merely 1 g of this indigoid [38]. Halogenations constitute a suitable leaving group to enable the synthesis of indigo copolymers used as semiconductor films [39].

**Figure 5:**
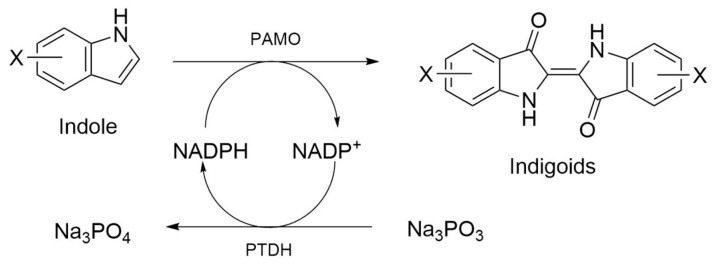
Scheme of the system to oxidize substituted indole into functionalized indigoids, using PAMO and phosphite dehydrogenase as a cofactor (NADPH) regenerator.

To test the substrate scope of PAMO_HPCD_ and PAMO_HPED_ for alkylated or halogenated indoles, we performed enzyme activity assays against indole and substituted indoles using crude extracts of *E. coli* cells overexpressing either enzyme, which were supplemented with phosphite dehydrogenase and its corresponding substrate to maintain the availability of NADPH during the reaction (Figure 5) [40,41]. These indole derivatives contain different groups, from methyl substituents to bigger halogenated heteroatoms, such as iodine, on C5 or C6 (Table 2).

In the presence of the novel PAMO variants, coloration was observed from deep blue to purple (Figure 6 and Figure S6) after overnight incubation in the substituted substrates (Table 2). The supernatant for each solution was further analyzed by high-resolution mass spectrometry to determine the reaction products (Figures S7–S16). Both enzymes utilized most of the C5- and C6-alkylated and halogenated indoles.

Importantly, only PAMO_HPCD_ catalyzed the production of Tyrian purple using 6-bromoindole as substrate. Recent developments for the biosynthetic production in *E. coli* of this indigoid dye from tryptophan designed a consecutive two-cell reaction system, where one of the bacteria produces halogenated tryptophan and the second bacteria overexpresses tryptophanase and a flavin monooxygenase for the oxidation of the halogenated tryptophan and the final production of Tyrian purple [42]. In this regard, PAMO_HPCD_ could also be employed to produce Tyrian purple in these biosynthetic two-cell reactions.

### 2.4. Steady-State Kinetic Parameters of the PAMO Variants

Both designed PAMO variants have shown activity using oxidizable substrates as indole, as well as many halogenated and alkylated derivatives, but a few differences were observed in both whole-cell biocatalysis and substrate scope assays. First, the production of indigo is much higher for *E. coli* cells overexpressing PAMO_HPCD_ than PAMO_HPED_. Second, only HPCD can produce Tyrian purple from halogenated 6-bromoindole.

Reasoning that these features are not due to differences in the protein expression yields for each enzyme but attributable to differences in their catalytic activity and catalytic site, we performed steady-state kinetic assays of NADPH depletion [23], in which the depletion in the presence of indole is measured spectrophotometrically at 341 nm. It is worth noting that NAPDH depletion can occur even in the absence of the indole substrate, which is why we used PAMO_WT_ as control.

From these assays, the kinetic parameters for each enzyme were determined, as summarized in Table 3. For enzyme kinetic assays as a function of increasing concentrations of NADPH, it is observed that the PAMO variants have similar Michaelis constants (*K_m_*) for this substrate, which are 5- to 10-fold higher than the *K_m_* for PAMO_WT_. In contrast, when these experiments are performed as a function of increasing concentrations of indole, the *K_m_* for PAMO_HPCD_ is half of that obtained for PAMO_HPED_. Additionally, PAMO_WT_ does not utilize indole as substrate. Moreover, the catalytic constant (*k*_cat_) for PAMO_HPCD_ is similar to PAMO_WT_, but it is 3-fold higher than the *k*_cat_ for PAMO_HPED_. Overall, the catalytic efficiency, expressed in terms of the ratio *k*_cat_/*K_m_*, is ~6 times higher for PAMO_HPCD_ over PAMO_HPED_, consistent with the differences in indigo production in whole-cell biocatalysis.

## 3. Discussion

The PAMO variants described herein, PAMO_HPCD_ and PAMO_HPED_, were determined to accept indole as substrate in *E. coli* cells to produce indigoids, given the change in coloration of the cell culture medium. This change was not observed in the PAMO_WT_ medium, even with long cell culture times. Some of the amino acid substitutions contained in these variants occur on residues that are part of loops of the FAD-binding domain and next to residue R337 in the active site, which were previously modified to increase the catalytic rate of PAMO against cyclohexanone as substrate [30]. Similar substitutions now allow these PAMO variants to produce indigo and indirubin.

Both a higher yield of indigo production in whole-cell biocatalysis and a higher *k*_cat_ was determined for PAMO_HPCD_, which only differs from PAMO_HPED_ in the amino acid on residue position 443. The indigo production yield in *E. coli* overexpressing PAMO_HPCD_, which is 3-fold higher than the 1.00 g/L production obtained using a flavin monooxygenase from *M. aminisulfidivorans* [35] without requiring supplementation of the cell medium with indole or L-tryptophan, propose this PAMO variant as a suitable biocatalyst.

Additionally, screening assays demonstrated that both PAMO variants can also incorporate indole derivatives with large halogen heteroatoms and alkylations, with only a few of the tested substrates not being accepted. We argue that this is due to electronic and structural characteristics of these derivatives, such as charges in the nitroindole compound, too large heteroatoms such as iodine, or the highly reactive warhead of the indole-5-carboxaldehyde. Importantly, only PAMO_HPCD_ was capable of synthesizing Tyrian purple, for which a whole-cell biosynthetic production in *E. coli* was recently described [42]. Thus, PAMO_HPCD_ can be further tested in these whole-cell biocatalysis platforms assessing potential increases in the production yield of this industrially relevant indigoid.

## 4. Materials and Methods

### 4.1. Chemical and Reagents

All chemical reagents used in this study were of analytical grade or higher. Indigo, indole, indirubin, indigo carmine, and L-tryptophan were purchased from AK Scientific (Ahern Avenue Union City, CA, USA). Terrific broth medium (TB) and L-arabinose were purchased from Thermo Fisher (Waltham, MA, USA). Solvents for HPLC analysis and the KOD Hot Start DNA polymerase were purchased from Merck KGaA (Darmstadt, Germany). DpnI restriction enzyme was purchased from New England Biolabs GmbH (Frankfurt am Main, Germany).

### 4.2. Creation of PAMO Variants

Plasmid pPAMO_PAC, derived from pPAMO [25] and carrying three substitutions in the protein sequence (P440F [43], Q93N, P94D [27]), was used as a template to create a saturation mutagenesis library for residues 441–444 by randomizing 2 amino acids using the QuikChange PCR method [44]. The codon degeneracy used was a mixture of NDT, VHG, and TGG. The mixing ratio of the primers was described in previous works [45]. The amplification reaction (20 µL) contained 10X KOD Buffer, dNTPs (2 mM each), MgCl_2_ (25 mM), mutagenic primers (3.5 µM each), template plasmid (50 ng), and KOD Hot Start polymerase (0.5 U). The PCR conditions were 1 cycle at 95 °C for 3 min; 27 cycles at 95 °C for 60 s, 55–65 °C (annealing temperature depending on the set of primers) for 60 s, 68 °C for 8 min; and a final additional extension step at 68 °C for 16 min. To hydrolyze the template plasmid, PCR products were directly digested with 1 µL of DpnI at 37 °C for 1.5 h, then another 1 µL DpnI was added to the reaction, and the incubation was continued for 90 more minutes. An aliquot of 2 µL was used directly to transform 50 µL of *E. coli* TOP10 chemo-competent cells by thermal shock. The transformation mixture was incubated with 1 mL of LB medium at 37 °C with shaking. After 1 h, 30 µL was spread on LB agar plates supplemented with 100 µg/mL carbenicillin (CB). PAMO variants found to serendipitously produce a blue coloration in cell cultures of the transformed *E. coli* TOP10 bacteria on LB medium were sequenced and subjected to further analysis. These enzymes correspond to PAMO_HPCD_ (Q93N, P94D, P440F, S441H, A442P, L443C, S444D) and PAMO_HPED_ (Q93N, P94D, P440F, S441H, A442P, L443E, S444D).

### 4.3. Enzyme Characterization

Enzymes (PAMO_WT_, PAMO_HPCD_, PAMO_HPED_) were purified from 500 mL cell culture in TB medium, inoculated with a 5 mL preinoculum of the corresponding plasmid-harboring bacteria in LB media that were obtained by overnight incubation at 37 °C. Overexpression was induced by adding L-arabinose to a final concentration of 2 mg·mL^−1^ upon reaching an optical density at 600 nm (OD_600_) of 0.6, followed by overnight incubation at 37 °C. Cells were then sedimented by centrifugation (20 min, 5000 rpm) and resuspended in 50 mM phosphate buffer pH 8.0 containing 1 mM phenylmethanesulfonyl fluoride (PMSF) protease inhibitor, after which they were lysed on a sonicator (12 cycles of 20 s ON, 40 s OFF, 40% amplitude) keeping the tube in an ice bath.

The lysate was centrifuged at 13,000 rpm and then filtered with 0.22 µM syringe filters before loading them onto a 5 mL HisTrap column (Cytiva, Marlborough, MA, USA), followed by elution using an increasing imidazole gradient from 20 to 100 mM on a buffer containing 50 mM phosphate buffer pH 8.0, 500 mM NaCl, and 10% glycerol. The purified enzymes can be detected by a strong yellow color due to the covalent-bound flavin adenine dinucleotide (FAD). The homogeneity of the enzyme solution was determined by SDS-PAGE.

### 4.4. Thermostability

The thermostability of PAMO_HPCD_ and PAMO_HPED_ was compared to PAMO_WT_ by differential scanning calorimetry (Nano DSC, TA Instrument, New Castle, DE, USA), where the melting temperature (Tm) necessary to unfold each enzyme was determined, using the same protein concentration for each enzyme. The Tm was determined by fitting the molar heat capacity data as a function of temperature to a two-state temperature unfolding model.

### 4.5. Indigo Production Using E. coli Expressing PAMO Variants

Cells producing PAMO_HPCD_ and PAMO_HPED_ were cultured in 5 mL of LB medium, and after 16 h of incubation at 37 °C, they were transferred to 50 mL of TB medium. Whole-cell reactions were initiated by adding L-arabinose solution (final concentration 2 mg·mL^−1^) to the medium. Whole-cell production proceeded at 37 °C for 48 h in a high-speed incubator (180 rpm), after which the reaction was quenched by centrifugation and resuspension in an equal volume of ethyl acetate, followed by vigorous mixing. The mixtures were then centrifuged at 13,000 rpm for 10 min, after which the blue-colored organic solvent layer containing indigo and indirubin was separated.

### 4.6. Optimization of the Whole-Cell Reaction

To optimize the reaction, different variables such as time, temperature, L-tryptophan, and indole concentrations were tested. The same culture method at two different temperatures, 30 °C and 37 °C, was performed, taking samples at 4, 8, 12, 16, 24, 48, and 72 h and measuring the production yields for indigo and indirubin. Once we determined the optimal time and temperature, the concentration of supplements such as indole and L-tryptophan were determined in the same way, with concentrations of 1, 10, and 20 µM for indole and 10, 100, and 200 µM for L-tryptophan.

### 4.7. Structural and Quantitative Analysis of Biosynthetically Produced Indigo

The fractions collected from the indigo-producing cell culture were separated by centrifugation and further subjected to thin layer chromatography (TLC) and HPLC analysis. For TLC analysis, the mobile phase was composed of hexane:ethyl acetate (1:1). The indigo and indirubin standard solutions were prepared by dissolving synthetic compounds in DMSO (0.1 to 100 ppm). Real sample indigoids products were standardized by dry cell weight (DCW) and then resuspended in acetonitrile (ACN). For the quantification, the samples were diluted 1:10 and injected into a HPLC (LC-4000 UV/Vis, JASCO corporation, Japan) equipped with a C18 reverse-phase column (Inertsil-C18 GL Sciences Inc., 250 mm, 4.6 mm, 3.5 mm, Tokyo, Japan) and eluted at 1.0 mL·min^−1^ with ACN/water (50:50 *v*/*v*) as mobile phase. The absorbance of the eluent was monitored using the JASCO UV-4075 UV/Vis dual absorbance detector at 268 and 293 nm. The production yield of indigo and indirubin was further determined using a standard calibration curve obtained using the same quantification methods with commercially available synthetic indigo and indirubin (Figure S4). In addition, the collected indigoids were analyzed by HRMS for further confirmation of the obtained products.

### 4.8. Steady-State Kinetics

Kinetic assays were performed in triplicates on a UV–Vis spectrophotometer (JASCO V-730, JASCO corporation, Tokyo, Japan), monitoring the change in absorbance at 341 nm to determine the depletion of the cofactor NADPH. Experiments were performed in a mixture containing 50 mM phosphate buffer pH 8.0, 1 µM freshly purified enzyme, and variable concentrations of NADPH (5.0 to 500 µM) and indole (0.05 to 1 mM), leaving one of the substrates fixed at saturating concentrations for the determination of the kinetic parameters for each substrate.

Kinetic parameters V_max_ and *K_m_* for each enzyme were determined with the kinetic module of the JASCO Spectra Manager suite (JASCO Corporation), using a Michaelis–Menten model. Then, *k*_cat_ and the catalytic efficiency (*k*_cat_/*K_m_*) were determined from this data.

### 4.9. Substituted Indoles Assay

This assay was performed to evaluate if the PAMO variants accept indoles with substituents in positions 5 or 6 of the heterocyclic ring. The substituents, either small alkanes or large halide atoms, are shown in Table 2.

The assays were performed using clarified crude extracts of *E. coli* cell cultures overexpressing the PAMO variants (total protein concentration ~5 µM), completing a 5 mL mixture containing 50 mM phosphate buffer pH 8.0, 10 µM phosphite dehydrogenase, 10 mM sodium phosphite, 200 µM NADPH, and 2 mM substituted indole. The reactions were carried out under vigorous shaking at 37 °C for 18 h, and then quenched by adding ethyl acetate to extract the organic compounds.

The reaction products were analyzed in a high-resolution mass spectrometer (Exactive Plus Orbitrap, Thermo Fisher Scientific, Bremen, Germany), using the following scan parameters: resolution: 140,000; AGC target: 1 × 10^6^; max. inject time: 200; HESI source: sheath gas flow: 15; aux gas flow rate: 5; sweep gas flow rate: 0; capillary temp: 250 °C; S-lens RF level: 100; heater temp: 100 °C; negative polarity; ionization voltage: 4 kV.

## 5. Conclusions

We developed monooxygenase enzymes through iterative saturation mutagenesis that was able to spread the scope of accepted substrates. They were obtained from *T. fusca* and called PAMO_HPCD_ and PAMO_HPED_ due to substituted residues (441–444), and then expressed in *E. coli* TOP10 to characterize the enzyme and their biocatalytic characteristics. These two new variant enzymes were serendipitously able to produce indigo in a rich media by L-tryptophan metabolism. Through HPLC analysis, we could identify two indigoids, indigo and indirubin, which have different means of production with each enzyme. With bibliographic reviews, we performed an optimization of the production by adding supplements to the media, such as indole or L-tryptophan. The highest productive yields of indigo were achieved by the addition of L-tryptophan to the culture media, which boosted indigo production to ~3.00 g/L. The indirubin productions of PAMO_HPCD_ and PAMO_HPED_ are close to 0.13 g/L of cell culture. Additionally, we explored the substrate acceptance of these variant enzymes, and they were shown to be able to oxidize almost all indole derivatives assayed.

## Figures and Tables

**Figure 6:**
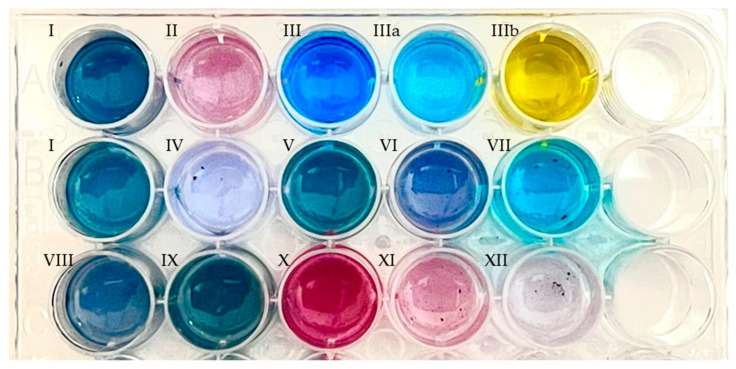
Indigoid derivatives, obtained by enzyme catalysis of the substrates indicated in Table 2 using clarified crude extracts of *E. coli* cell cultures overexpressing PAMO_HPCD_, showing their color shifts due to their substitutions. Enzymatic reactions were performed in phosphate buffer supplemented with phosphite dehydrogenase and sodium phosphate for cofactor regeneration. Then, the products were extracted in ethyl acetate and resuspended in DMSO. The first row corresponds to commercial indigoid standards. First row: I, indigo; II, indirubin; III, indigo carmine (water); IIIa, indigo carmine (HCl 0.10 N); IIIb, indigo carmine (NaOH 0.10 M). Second row: I, indigo; IV, 5,5′-dicyanoindigo; V, 5,5′-difluoroindigo; VI, 5,5′-dichloroindigo; VII, 5,5′-hydroxyindigo. Third row: VIII, dimethylindigo; IX, 5,5′-dimethoxyindigo; X, 6,6′-difluoroindigo; XI, 6,6′-dichloroindigo; XII, 6,6′-dibromoindigo.

**Table 1:** Production of indigo and indirubin by whole-cell biocatalysis using *E. coli* cells overexpressing PAMO_HPCD_ and PAMO_HPED_ under different cell culture conditions.

	HPCD	HPCD_indole_	HPCD_tryptophan_	HPED	HPED_indole_	HPED_tryptophan_
Indigo (mg/g DCW)	0.388 ± 0.009	0.484 ± 0.125	0.498 ± 0.024	0.015 ± 0.006	0.007 ± 0.002	0.016 ± 0.007
Indirubin (mg/g DCW)	0.009 ± 0.001	0.013 ± 0.001	0.019 ± 0.001	0.008 ± 0.002	0.009 ± 0.001	0.016 ± 0.001

**Table 2:** Substrates tested in the new enzymes and confirmed through exact mass in HRMS assay.

Substrate	HPCD	HPED	Product *	Exact Mass (g mol^−1^)	Product Nomenclature
Indole	+	+	Indigo	262.0742	I
5-Cyanoindole	+	+	5,5′-dicyanoindigo	312.0647	IV
5-Fluroindole	+	+	5,5′-difluoroindigo	298.0554	V
5-Chloroindole	+	+	5,5′-dichloroindigo	329.9963	VI
5-Hidroxyindol	+	+	5,5′-hydroxyindigo	296.0641	VII
5-Methylindol	+	+	5,5′-dimethylindigo	290.1055	VIII
5-Methoxyindol	+	+	5,5′-dimethoxyindigo	322.0954	IX
6-Fluoroindole	+	+	6,6′-difluoroindigo	298.0554	X
6-Chloroindole	+	+	6,6′-dichloroindigo	329.9963	XI
6-Bromoindole	+	−	6,6′-dibromoindigo	417.8953	XII
5-Nitroindole	−	−	-	-	-
5-Iodoindole	−	−	-	-	-
5-(Benzyloxy)Indole	−	−	-	-	-
Indole-5-carboxaldehyde	−	−	-	-	-

* HRMS spectra of the indigo derivatives in Figure S6.

**Table 3:** Kinetic parameters obtained for PAMO and both variants.

	NADPH			Indole		
Variant	*k*_cat_ (s^−1^)	*K_m_* (µM)	*k*_cat_/*K_m_* (s^−1^M^−1^)	*k*_cat_ (s^−1^)	*K_m_* (μM)	*k*_cat_/*K_m_* (s^−1^M^−1^)
PAMO_WT_	4.8	0.9	5,200,000	-	-	-
PAMO_HPCD_	6.8	7.6	900,000	5.4	58.2	93,000
PAMO_HPED_	1.9	4.4	430,000	1.7	109.0	16,000

## Data Availability

Not applicable.

## Supplementary Materials

The following supporting information can be downloaded at: https://www.mdpi.com/article/10.3390/ijms232012544/s1.
