# Supplementary material for: Discovery of New Phenylacetone Monooxygenase Variants for the Development of Substituted Indigoids through Biocatalysis
Source: Int J Mol Sci. 2022 Oct 19;23(20):12544. doi: 10.3390/ijms232012544 (PMC9604523; doi:10.3390/ijms232012544)
Supplement: Supplementary file 1 [file ijms-23-12544-s001.zip › ijms-1969954-supplementary.pdf]

Supplementary Materials

Discovery of new phenylacetone monooxygenase variants for the development of substituted indigoids through biocatalysis

Nicolás Núñez-Navarro[1,2,3], Javier Salazar Muñoz[1,4], Francisco Castillo[1], César A. Ramírez-Sarmiento[2,5], Ignacio Poblete-Castro[6], Flavia C. Zacconi[1,2,4,*], Loreto P. Parra[2,3,*],

[1]Facultad de Química y de Farmacia, Pontificia Universidad Católica de Chile, Santiago, Chile
[2]Institute for Biological and Medical Engineering, Schools of Engineering, Medicine and Biological Sciences, Pontificia Universidad Católica de Chile, Santiago, Chile.
[3]Department of Chemical and Bioprocesses Engineering, School of Engineering, Pontificia Universidad Católica de Chile, Santiago, Chile
[4]Center for Nanomedicine, Diagnostic & Drug Development (ND3), Universidad de Talca, Talca 3460000, Chile
[5] ANID – Millennium Science Initiative Program – Millennium Institute for Integrative Biology (iBio), Santiago, Chile
[6] Biosystems Engineering Laboratory, Department of Chemical and Bioprocess Engineering, Universidad de Santiago de Chile (USACH), Santiago, Chile
Correspondence: lparrat@uc.cl (L.P.P.); fzacconi@uc.cl (F.C.Z.).

Table of contents

1. **Production of indigo and indirubin in recombinant *E. coli*.** 2
2. **Sequence analysis of $PAMO_{WT}$ and its variants** 3
3. **Electrophoretic mobility analysis of $PAMO_{WT}$ and its variants** 4
4. **Thermal stability of $PAMO_{WT}$, $PAMO_{HPCD}$ and $PAMO_{HPED}$ determined by DSC** 5
5. **Standardization of HPLC-UV/Vis for indigo and indirubin concentration** 6
6. **Pigment patron obtained with PAMO variants** 7
7. **High-resolution mass spectrometry (HRMS) of indigo derivatives** 8

1. **Production of indigo and indirubin in recombinant *E. coli*.**

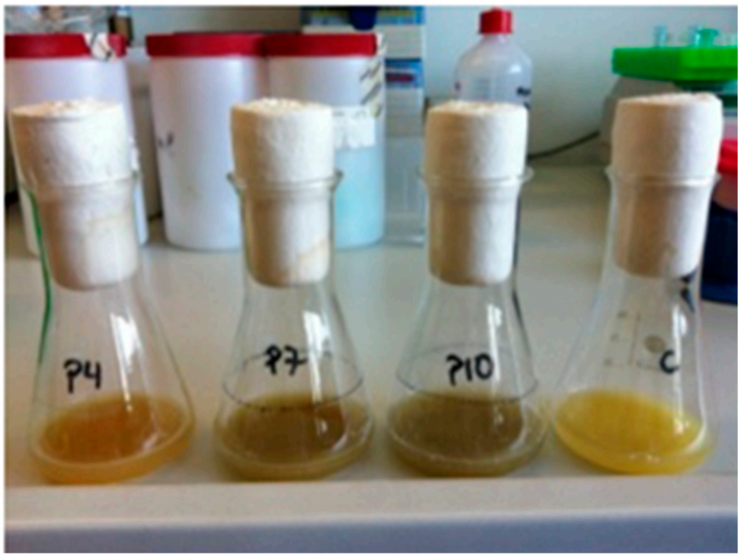

Figure S1. Production of indigo and indirubin in recombinant *E. coli* overexpressing $PAMO_{HPCD}$ or $PAMO_{HPED}$. The formation of these pigments can be seen by comparing the C flask (*E. coli* overexpressing $PAMO_{WT}$) with *E. coli* overexpressing $PAMO_{HPCD}$ in flask P7 and P10, and $PAMO_{HPED}$ in flask P4.

2. Sequence analysis of PAMO$_{WT}$ and its variants

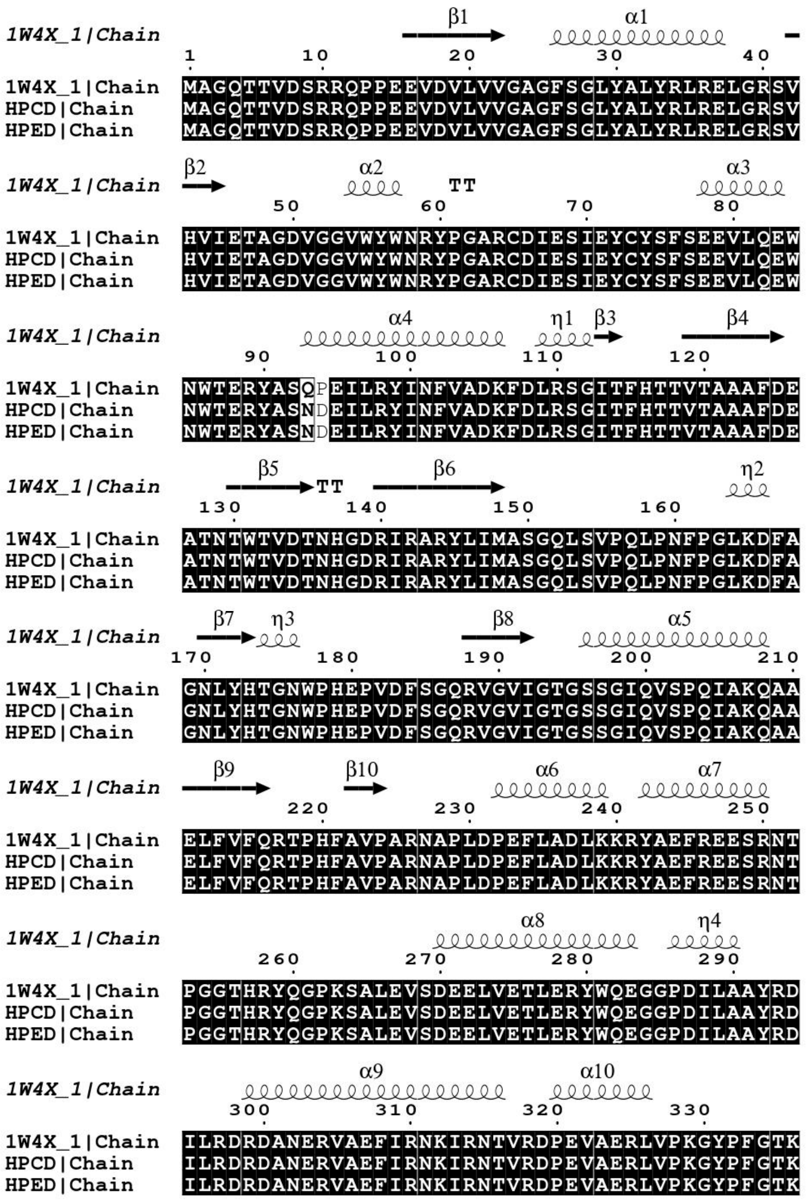

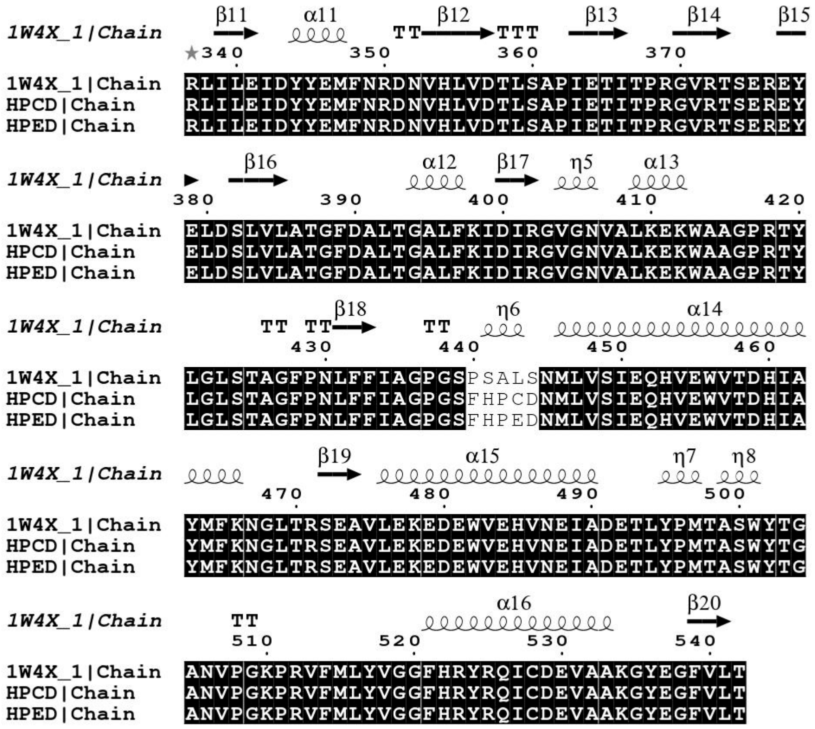

Figure S2. Sequence alignment of PAMO$_{WT}$ and its new variants PAMO$_{HPCD}$ and PAMO$_{HPED}$. Black boxes correspond to the conserved residues in all sequences, whereas white boxes indicate the residues that have been substituted.

3. Electrophoretic mobility analysis of PAMO$_{WT}$ and its variants

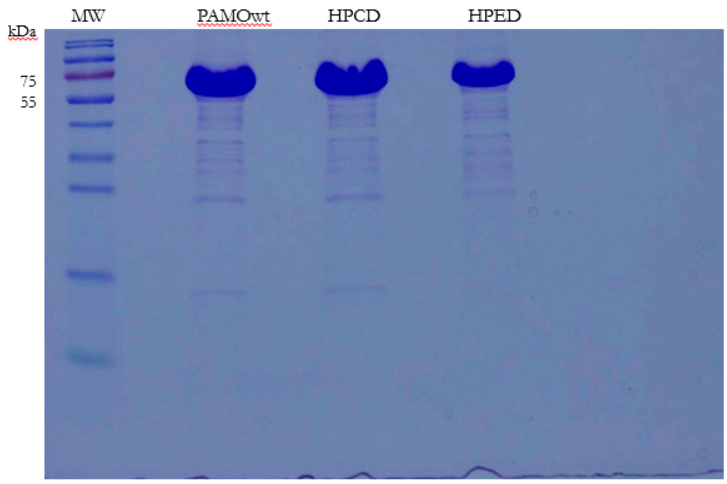

Figure S3. SDS-PAGE of the purified enzymes PAMO$_{WT}$ and the new variants PAMO$_{HPCD}$ and PAMO$_{HPED}$. The enzymes, which have molecular weight of ~62 kDa, are highly pure and visible in the gap between 55 and 75 kDa of the protein ladder.

4. **Thermal stability of PAMOWT, PAMOHPCD and PAMOHPED determined by DSC**

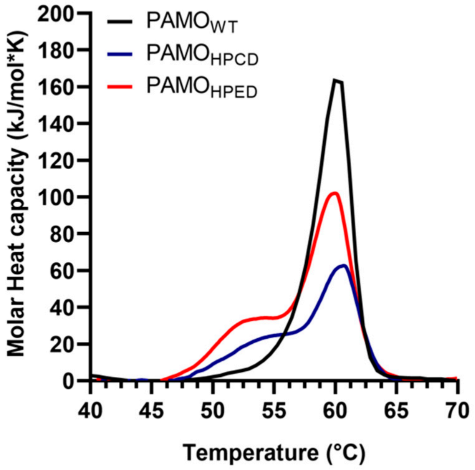

Figure S4. Thermal stability of PAMOWT, PAMOHPCD and PAMOHPED determined by DSC. The Tm of each enzyme corresponds to the temperature in which the molar heat capacity reaches its maximum value.

5. **Standardization of HPLC-UV/Vis for indigo and indirubin concentration**

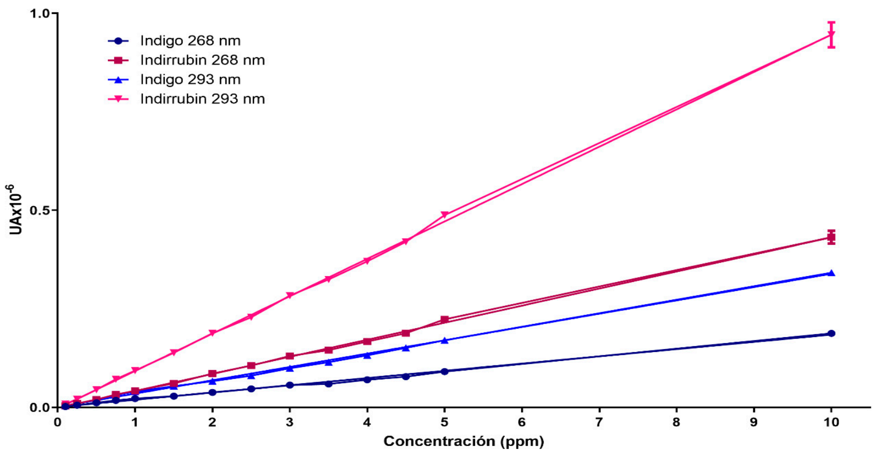

Figure S5. Calibration curves for HPLC-UV/Vis.

The calibration curve for HPLC with the standard indigo and indirubin was carried out in a concentration range between 0.1 ppm to 10 ppm with 20 µL of injection.

6. Pigment patron obtained with PAMO variants

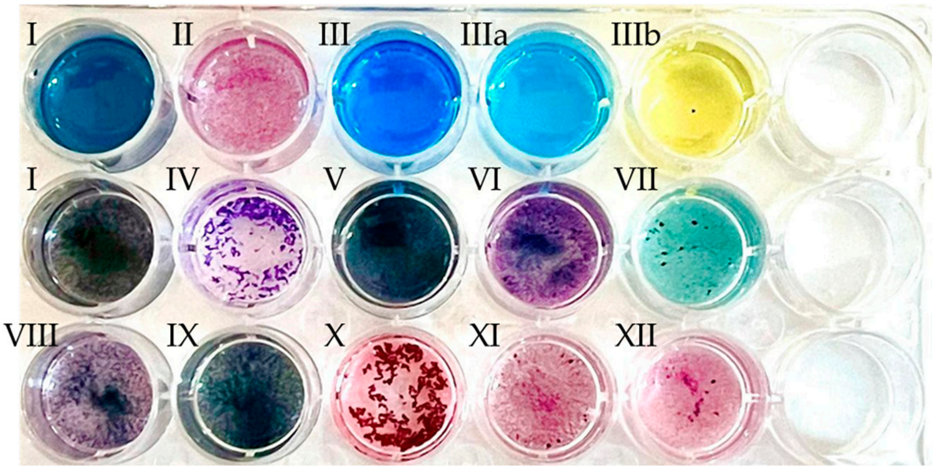

Figure S6. Indigoid derivatives obtained by enzyme catalysis of the substrates indicated in Table 2, using clarified crude extracts of *E. coli* cell cultures overexpressing $PAMO_{HPCD}$ and supplemented with phosphite dehydrogenase and sodium phosphite for cofactor regeneration. This figure resembles Figure 6 in the main text, with the exception that the indigo derivatives obtained by enzyme catalysis were kept in phosphate buffer, in which most of them are insoluble solids as it is clear from this image. The first row corresponds to commercial indigoid standards. First row: I, indigo (DMSO); II, indirubin (DMSO); III, indigo carmine (water); IIIa, indigo carmine (HCl 0.10 N); IIIb, indigo carmine (NaOH 0.10 M). Second row: I, indigo; IV, 5,5′-dicyanoindigo; V, 5,5′-difluoroindigo; VI, 5,5′-dichloroindigo; VII, 5,5′-hydroxyindigo. Third row: VIII, dimethylindigo; IX, 5,5′-dimethoxyindigo; X, 6,6′-difluoroindigo; XI, 6,6′-dichloroindigo; XII, 6,6′-dibromoindigo.

7. High-resolution mass spectrometry (HRMS) of indigo derivatives. Negative ion spectra.

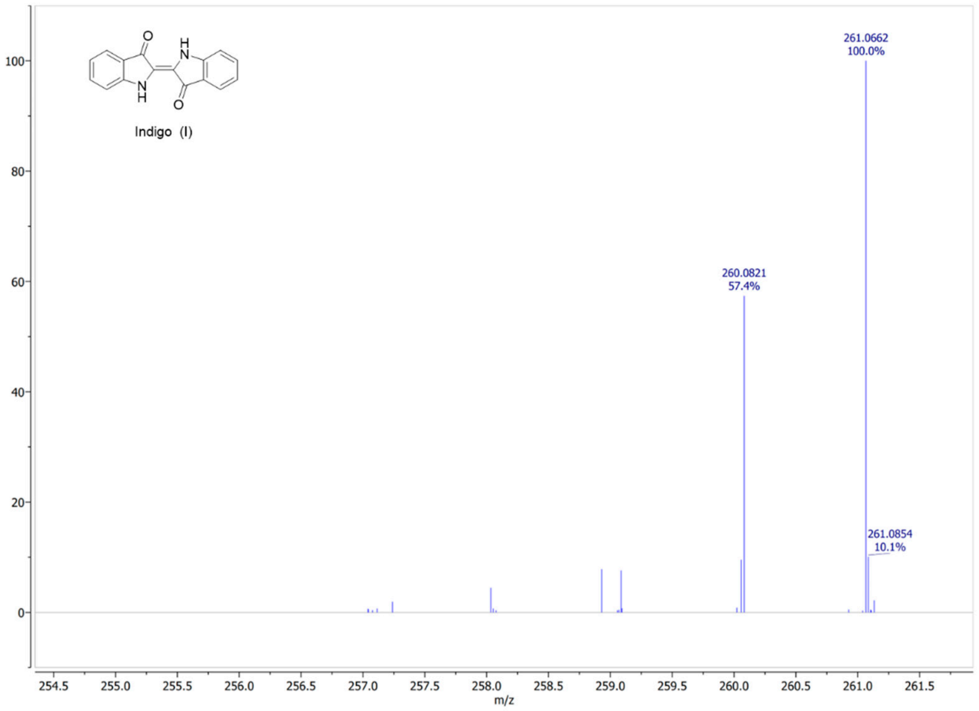

Figure S7. HRMS of indigo (I). $[M-H]^-$ = 261.0662 (100%)

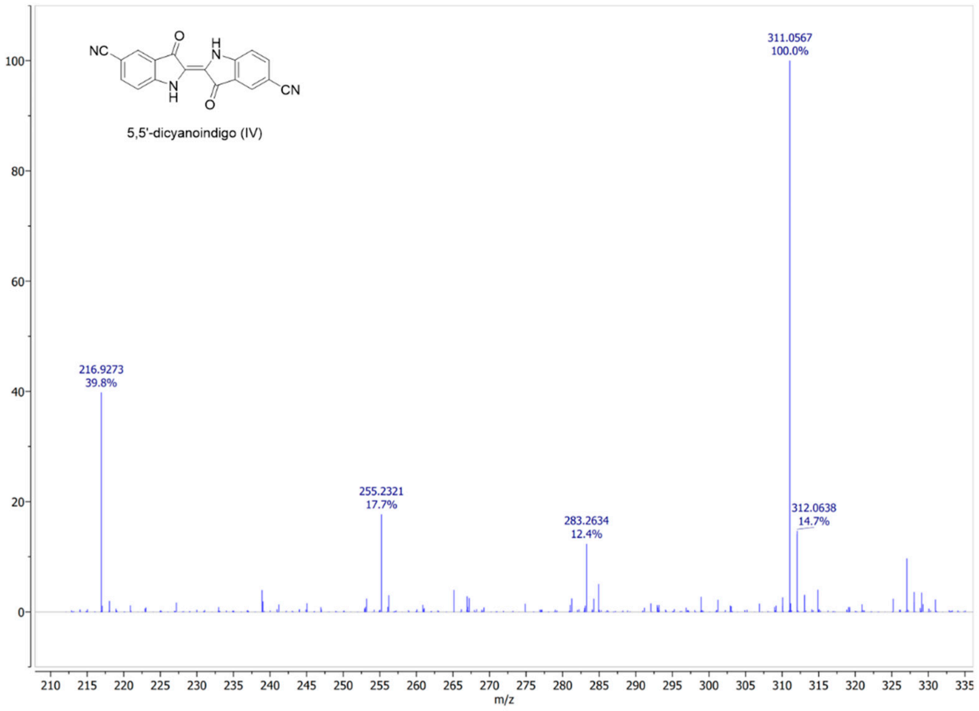

Figure S8. HRMS of 5,5′-dicyanoindigo (IV). $[M-H]^-$ = 311.0567 (100%)

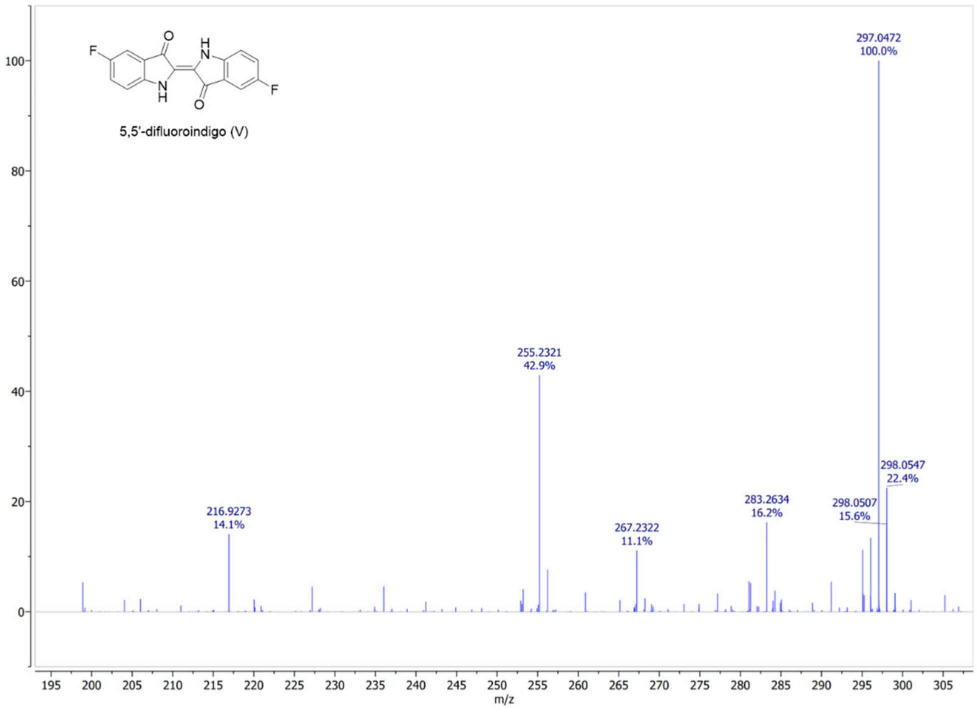

Figure S9. HRMS of 5,5′-difluoroindigo (V). [M-H]⁻ = 297.0472 (100%)

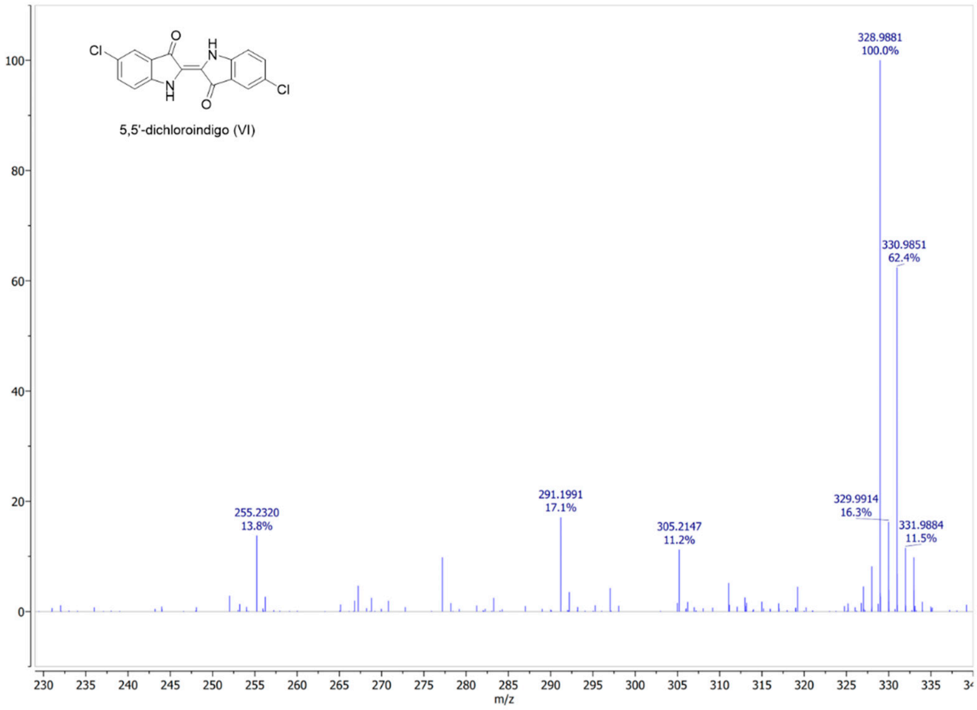

Figure S10. HRMS of 5,5′-dichloroindigo (VI). [M-H]⁻ = 328.9881; m/z = 329.9914 (16.3%); m/z = 330.9851 (62.4%); m/z = 331.9884 (11.5%)

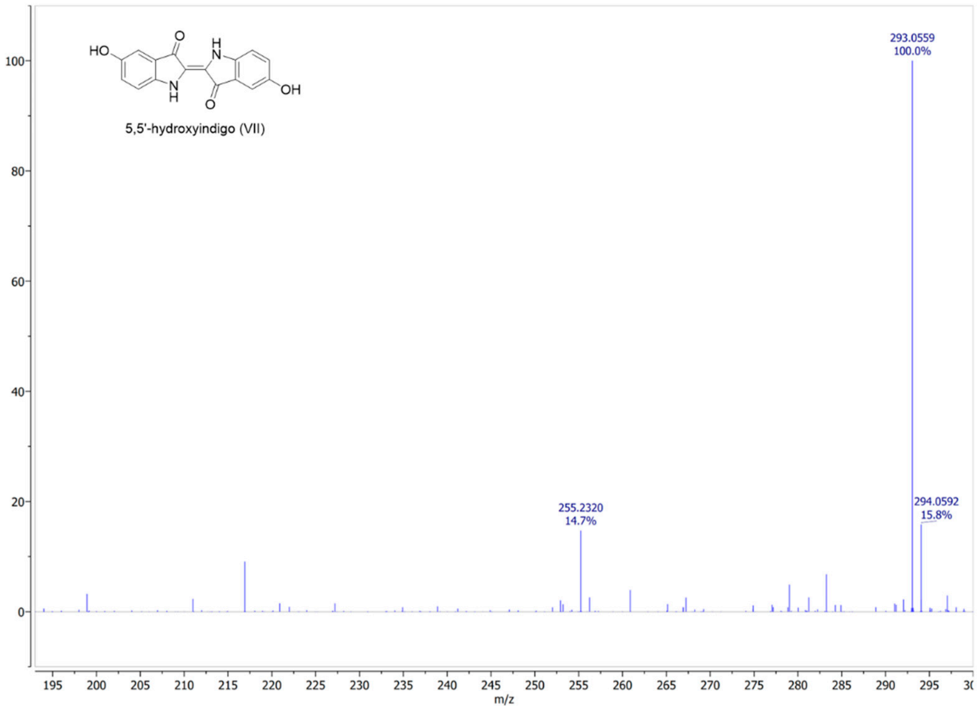

Figure S11. HRMS of 5,5′-hydroxyindigo (VII). $[M\text{-}H]^-$ = 293.0559 (100%)

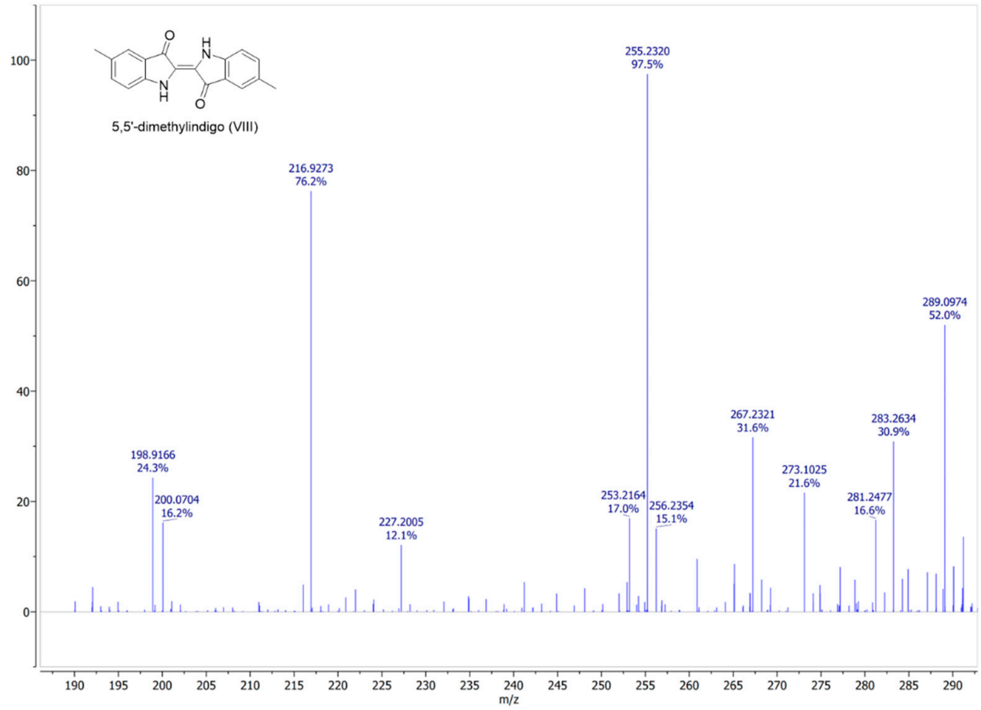

Figure S12. HRMS of 5,5′-dimethylindigo (VIII). $[M\text{-}H]^-$ = 289.0974 (52%)

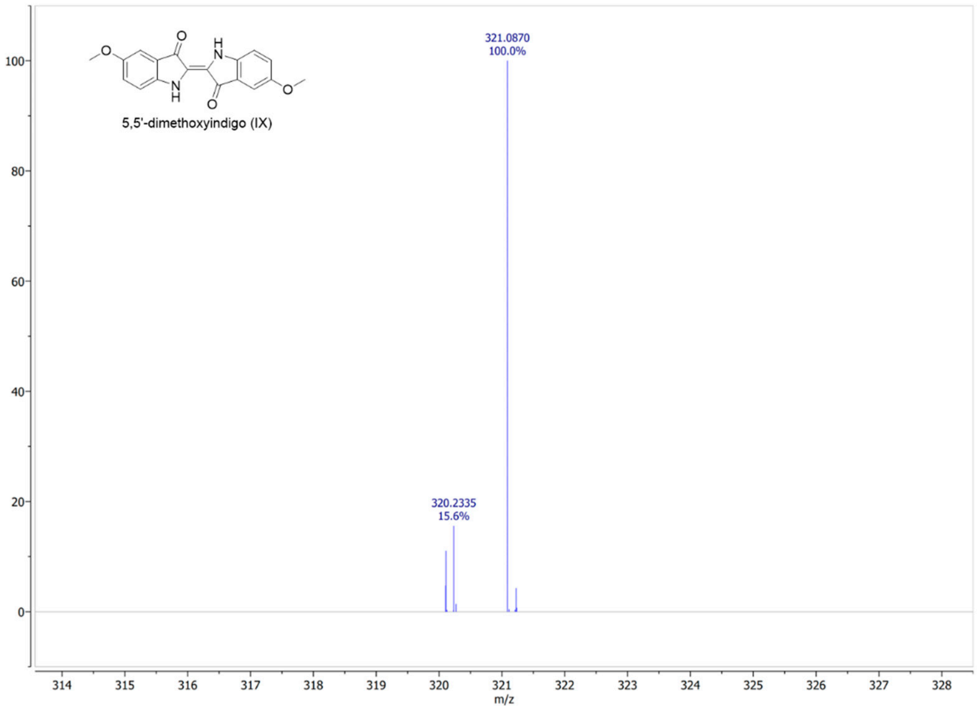

Figure S13. HRMS of 5,5′-dimethoxyindigo (IX). [M-H]$^-$ = 321.0870 (100%)

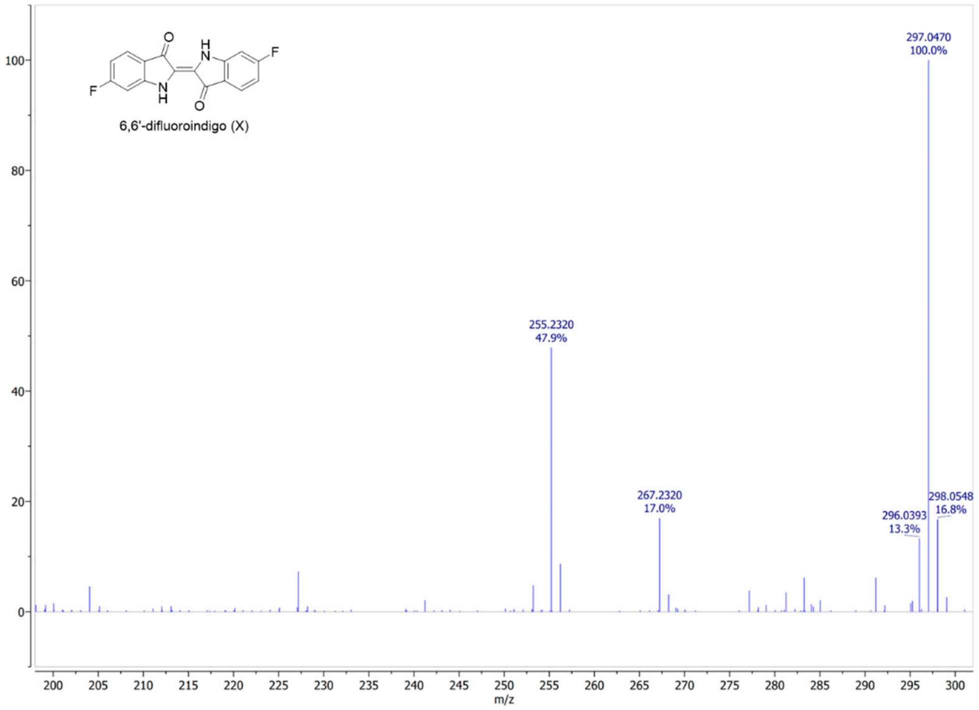

Figure S14. HRMS of 6,6′-difluoroindigo (X). [M-H]$^-$ = 297.0470 (100%)

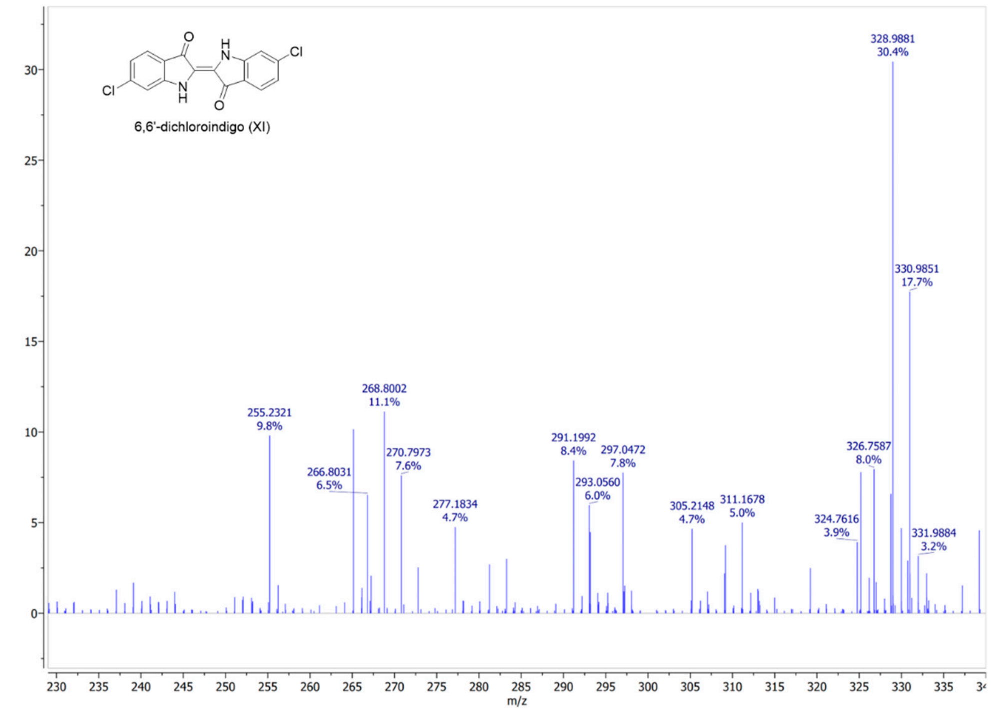

Figure S15. HRMS of 6,6′-dichloroindigo (XI). $[M\text{-}H]^-$ = 328.9881 (30.4%); m/z = 330.9851 (17.7%); m/z = 331.9884 (3.2%)

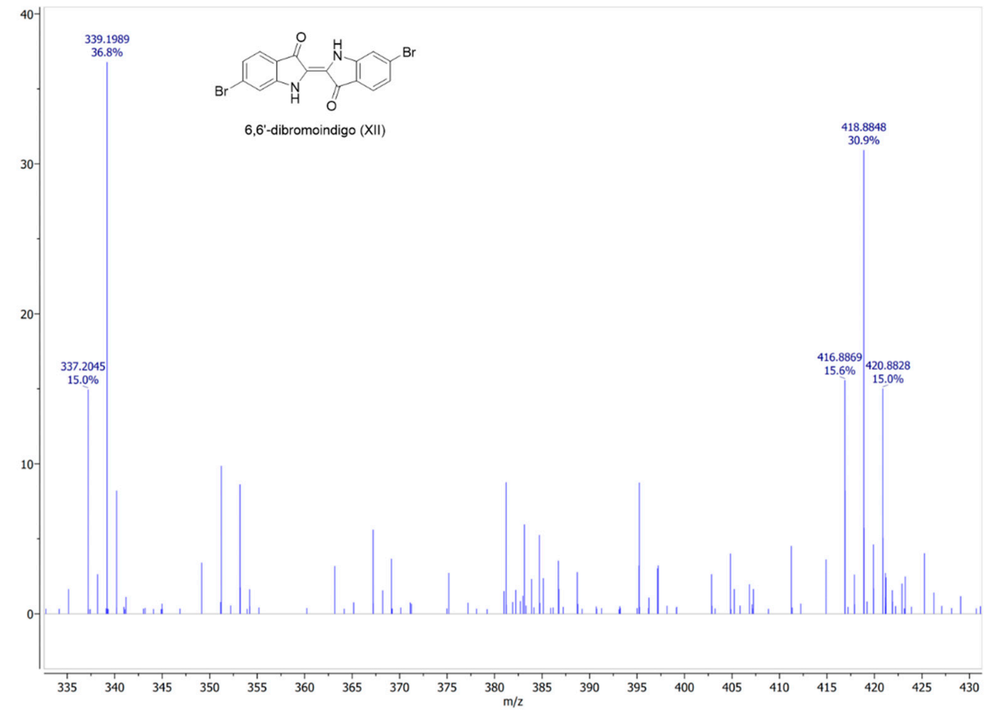

Figure S16. HRMS of 6,6′-dibromoindigo (XII). $[M\text{-}H]^-$ = 416.8869 (15.6%); m/z = 418.8848 (30.9%); m/z = 420.8828 (15%)
